# First-Mile Walking to Transit and Physical Activity: A Cross-Sectional Study of the MRT Pink Line Corridor in Bangkok, Thailand

**DOI:** 10.3390/ijerph23060810

**Published:** 2026-06-18

**Authors:** Sigit D. Arifwidodo, Nattanon Ubontip, Natsiporn Sangyuan, Orana Chandrasiri, Panitat Ratanawichit, Putthipanya Rueangsom

**Affiliations:** 1 Department of Landscape Architecture, Faculty of Architecture, Kasetsart University, Bangkok 10900, Thailand; nattanon.u@ku.th (N.U.); natsiporn.sa@ku.th (N.S.); panitat.r@ku.th (P.R.); 2 Activethai Research Center, Faculty of Architecture, Kasetsart University, Bangkok 10900, Thailand; orana.chandrasiri@gmail.com (O.C.); p.rueangsom@gmail.com (P.R.)

**Keywords:** first-mile walking, transit-oriented development, perceived walkability, NEWS-A, physical activity, GPAQ, pedestrian infrastructure, perceived traffic safety, Bangkok

## Abstract

**Highlights:**

**Public health relevance—How does this work relate to a public health issue?**

- Walking to mass rapid transit is a routine source of physical activity that can help populations reach recommended activity levels.
- In tropical, car-oriented cities like Bangkok, poor sidewalks and unsafe traffic can suppress this walking, reducing both mobility and health benefits.

**Public health significance—Why is this work significant to public health?**

- Pedestrian infrastructure quality and traffic safety are the strongest environmental correlates of first-mile walking in a suburban Asian transit corridor.
- Testing physical activity outcomes with and without transport walking separates real behavioural association from measurement overlap in the walking–physical activity link.

**Public health implications—What are the key implications or messages for practitioners, policymakers and/or researchers in public health?**

- Transport and public health policy should prioritise continuous sidewalks, safer crossings, traffic calming, and shaded routes, especially in the 201–1000 m ring around stations.
- Studies of transit-related physical activity should report both total and non-transport activity to avoid overstating the health contribution of access walking.

**Abstract:**

*Background.* First-mile walking to mass rapid transit (MRT) has two methodological problems. Composite walkability scores blur which features drive walking. And because walking to transit is itself transport physical activity (PA), linking it to total PA is circular. Both issues are sharper in tropical Asian cities. *Methods.* We surveyed 378 adults within a 1 km network distance of 20 stations on Bangkok’s Pink Line MRT. Walkability was measured with NEWS-A (aggregate and eight subscales); PA with the GPAQ. Binary logistic regression with station-cluster-robust standard errors tested which NEWS-A subscales predict first-mile walking and whether walkers meet the WHO PA guideline (≥150 min/week MVPA). A tautology sensitivity test removed transport PA from the outcome. *Results.* Walkers were 71.7% of the sample. Disaggregating NEWS-A improved fit; two subscales were the dominant predictors: pedestrian infrastructure and traffic safety. Walkers were 30.6 percentage points more likely to meet the overall PA guideline; with transport PA removed, the gap was 17.5 points and still significant. The pedestrian infrastructure effect was strongest 201–1000 m from a station, not at the immediate frontage. *Conclusions.* Perceived pedestrian infrastructure quality and perceived traffic safety drive first-mile walking in suburban Bangkok. The walking–PA link is not entirely a measurement artefact. The 201–1000 m ring is a plausible priority for pedestrian investment.

## 1. Introduction

Every mass rapid transit (MRT) journey requires passengers to cover the distance between their home and the station. In the transport planning literature, this segment is known as the “first mile,” while the equivalent segment at the destination end is the “last mile” [1,2]. First-mile walking, defined here as walking from home to the nearest MRT station, is the most common access mode to transit worldwide [3,4]. When the first-mile route is too long, uncomfortable, or dangerous, people drive instead, and transit ridership declines [5,6]. The first-mile/last-mile (FM/LM) gap is therefore a well-documented barrier to public transit use and a central concern in transit-oriented development planning [7,8].

First-mile walking also has direct relevance to public health. Because it accumulates through daily routine rather than deliberate exercise, it represents a form of physical activity (PA) [9,10]. Systematic reviews report that public transit users walk 8–33 min more per day than private vehicle users, a margin that contributes substantially toward the World Health Organisation’s recommended ≥150 min/week of moderate-to-vigorous physical activity (MVPA) [11,12]. Cross-sectional studies in the United States, Australia, and Asia have found that adults who walk to transit are more likely to meet PA thresholds than those who drive [13,14,15]. First-mile walking is therefore a meaningful source of routine PA for residents of transit catchments, and promoting it is an investment in population health as much as in transit ridership.

Two broad categories of route-side factors influence whether residents walk to transit: proximity and the quality of the neighbourhood pedestrian environment. Proximity to the station is the most studied predictor, and shorter distances are consistently associated with higher walking rates [16,17]. Walking distance is itself not constant across transit modes: passengers walk further to access higher-quality services such as rail or MRT than to access lower-frequency bus stops, a service-supply effect that explains why rail catchments are conventionally drawn larger than bus-stop catchments [3,5]. However, proximity alone does not guarantee walking. The pedestrian environment along the route also matters, and it is commonly assessed using composite instruments such as the Neighbourhood Environment Walkability Scale–Abbreviated (NEWS-A) [18]. Higher aggregate NEWS-A scores are associated with greater utilitarian walking across both Western and Asian cities [19,20]. The NEWS-A captures eight subscales: residential density, land use diversity, land use access, street connectivity, walking infrastructure quality, aesthetics, traffic safety, and crime safety [21]. In transit catchments, the interplay between proximity and these walkability dimensions determines whether the opportunity created by a nearby station translates into actual walking behaviour [22].

Despite consistent evidence that both proximity and walkability predict first-mile walking, three methodological and empirical problems limit what the existing literature can tell us. The first is the aggregation problem. Most studies use a single composite walkability score, which collapses the eight NEWS-A subscales into one number [23,24]. This approach masks the possibility that individual dimensions pull in opposite directions. High land use diversity, for instance, is a feature of transit-oriented development but often coincides with heavy vehicular traffic and pedestrian conflict [25,26]. If a pro-walking dimension and an anti-walking dimension cancel each other out, the aggregate score appears moderate even though the underlying environment is polarised. Disaggregating the NEWS-A is necessary to identify which specific pedestrian environment features drive or suppress first-mile walking [27].

The second is the tautology problem. First-mile walking is classified as transport-domain PA within instruments like the Global Physical Activity Questionnaire (GPAQ), so correlating it with total PA sufficiency risks circularity: the predictor is partly contained in the outcome [28]. Unless the walking–PA association holds after transport-domain PA is removed, studies may overstate the health benefits of transit walking. This concern has been widely acknowledged but rarely tested empirically, including in a recent Bangkok study that reported an unadjusted OR of around six [29].

The third is geographic and climatic context. The majority of evidence on first-mile walking, walkability, and health comes from temperate Western cities [30,31]. Whether these relationships hold in tropical, high-density Asian megacities with different infrastructure standards, climate constraints, and traffic conditions is largely untested [32].

Bangkok is well suited for addressing all three problems. The city’s tropical climate imposes heat, humidity, and monsoon rainfall as practical barriers to walking [33,34,35]. Decades of car-oriented planning have produced uneven sidewalk coverage, frequent vendor encroachment on pedestrian space, and high-speed traffic on arterial roads [36]. These conditions mean that living near an MRT station does not guarantee a walkable route to it. The recent opening of the Pink Line monorail corridor (Khae Rai–Min Buri, 34.5 km, 30 stations, operational since 2023) provides a setting with wide variation in both station proximity and walking infrastructure quality. Unlike Bangkok’s older central corridors where walking patterns are established, the Pink Line passes through heterogeneous suburban landscapes where residents are still adapting their travel behaviour. Despite heavy public investment in this expansion, few studies have isolated the specific environmental features that determine whether residents walk to stations, and fewer still have tested whether that walking produces genuine health benefits once measurement tautology is accounted for.

This study examines first-mile walking to MRT stations along the Pink Line corridor through a cross-sectional survey of 378 adults within a 1 km network distance of 20 stations. The study addresses two research questions: (1) What built environment factors, including disaggregated neighbourhood walkability dimensions, predict first-mile walking to MRT stations? (2) Does first-mile walking to MRT stations contribute to meeting WHO-recommended physical activity guidelines (≥150 min/week MVPA), and does this association survive the removal of transport-domain physical activity from the outcome?

This study contributes in three ways. First, it disaggregates the NEWS-A and uses a likelihood ratio test to show which subscales independently predict first-mile walking, an approach that recovers information lost in composite walkability scores. Second, it introduces a tautology sensitivity test for the walking–PA association that separates mechanical overlap, the transport-domain PA contained within the total PA outcome, from residual behavioural association, and applies it to the Bangkok setting where previous work has reported large walking–PA estimates without this adjustment. Third, by stratifying walkability effects across inner, mid, and outer distance bands, it identifies where within a station catchment walking infrastructure investment is most likely to alter walking behaviour. Collectively, these contributions provide methodological steps that are replicable in other transit contexts and targeted evidence for urban planners in Bangkok and other dense, climatically challenging cities.

## 2. Materials and Methods

### 2.1. Study Design and Setting

A cross-sectional survey was conducted among adult residents living within a 1 km network isochrone of stations along the MRT Pink Line monorail corridor in Bangkok, Thailand. The Pink Line monorail (PK01–PK30) opened in 2023 and runs from Khae Rai in Nonthaburi province to Min Buri in eastern Bangkok. The corridor spans 34.5 km with 30 stations, connecting the northern suburbs of Nonthaburi Province to the eastern fringe of Bangkok.

**Figure 1:**
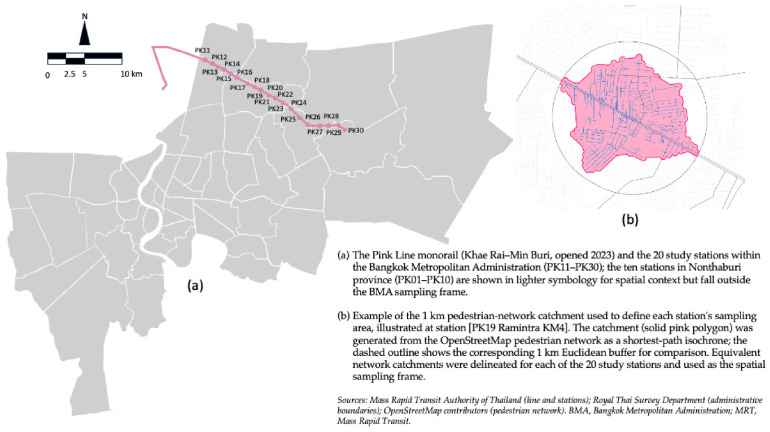
Study area and station catchment definition along the MRT Pink Line corridor, Bangkok.

**Figure 2:**
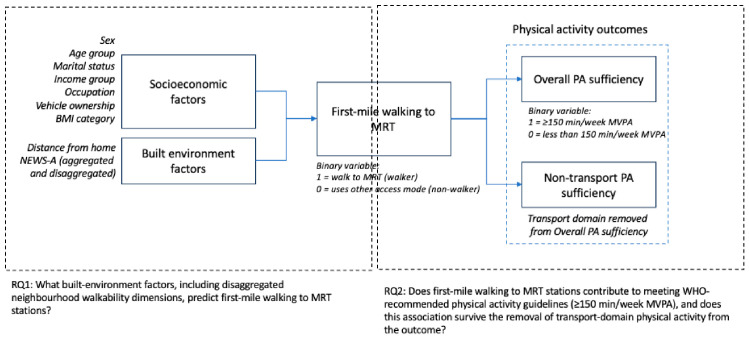
Conceptual diagram of the study.

Of the 30 stations, 20 fall within the Bangkok Metropolitan Administration (BMA) boundary. This study focuses on the 20 stations located within the Bangkok Metropolitan Administration (PK11–PK30), with each station defining a 1 km pedestrian network catchment that served as the spatial sampling frame (Figure 1). We restricted the sample to these 20 BMA stations to maintain administrative homogeneity in land use regulation, walking infrastructure provision, and public service delivery. The study corridor traverses a heterogeneous urban landscape, from dense commercial zones adjacent to major intersections to lower-density residential neighbourhoods at the periphery, and this variation in land use provides natural differences in both walking infrastructure quality and residential proximity to stations. The analytic structure of the study, including the predictors tested in RQ1 and the two outcome definitions used in RQ2, is shown in Figure 2.

The 1 km network isochrone was selected as the catchment boundary on both empirical and planning grounds. This threshold corresponds to approximately 10 to 12 min of walking for a healthy adult and exceeds the conventional 400 to 800 m planning standard [16,17]. However, it aligns with evidence on acceptable walking distances in high-density Asian urban contexts [36] and with evidence that passengers walk further to access higher-quality rail and MRT services than to access bus stops [3,5]. Because all 20 study stations belong to the same Pink Line corridor, operated by a single operator with the same rolling stock, service speed, and headway during normal operating hours, transit service quality is held constant across observations and does not confound the route-level walkability associations examined in this study. The threshold captures a broader population at varying levels of transit accessibility, which in turn allows detection of distance-gradient effects.

Network isochrones were generated for each of the 20 study stations using OpenStreetMap (OSM) pedestrian network data in QGIS LTR version 3.34.11-Prizren, accounting for actual street connectivity and physical barriers such as elevated roads, canals, and rail corridors that constrain pedestrian movement.

The study protocol was approved by the Institutional Review Board of Kasetsart University, Bangkhen Campus (COE No. COE68/110). All participants provided written informed consent prior to survey administration. Participation was voluntary and could be withdrawn at any time without consequence. No personally identifiable information was retained in the analytical dataset.

### 2.2. Participants and Sampling

Participants were recruited using a stratified random sampling design. The 20 Pink Line stations within BMA served as strata, with equal allocation of 20 respondents per station, yielding a target sample of 400. The station catchment was the natural sampling unit for four reasons. First, the catchment is the unit of policy interest, and equal allocation across the corridor covers the full range from dense commercial nodes to lower-density peripheries. Second, pedestrian environment conditions vary within station catchments at least as much as between them, and random sampling within each 1 km isochrone captures this within-catchment heterogeneity. Third, the NEWS-A walkability dimensions are the predictors of interest, and sampling on the predictor can bias predictor–outcome estimates; stratifying on an exogenous factor (station) and measuring exposure and outcome separately allows built environment variation to enter the analysis through NEWS-A rather than through the sampling frame. Fourth, perceived walkability is a respondent-reported measure, so using it as a sampling stratum would have required a corridor-wide objective audit that was not available at fieldwork.

The sampling procedure comprised three stages. First, the 1 km network isochrone was generated for each station, and all residential households within each isochrone were enumerated using GIS overlay of building footprint data and field verification. Second, 20 households were randomly selected from the enumerated list for each station. Third, one adult resident per selected household was invited to participate. Inclusion criteria required participants to be aged 18 years or older and to have resided at their current address for at least six months prior to the survey, so as to ensure familiarity with the local pedestrian environment. Residents who had relocated within the preceding six months were excluded.

Data were collected through face-to-face, paper-based interviews conducted between 15 and 23 November 2025 by a team of five trained surveyors, all graduate students in the Master of Landscape Architecture programme at Kasetsart University. Surveyors received standardised training on instrument administration, informed consent procedures, residential address geocoding, and the replacement sampling protocol. To reduce potential social desirability bias in self-reported physical activity (PA) and walking behaviour, surveyors were instructed to read questionnaire items verbatim, avoid leading prompts, and reassure participants that there were no correct or expected answers. Each interview required approximately 20 to 25 min. Respondents received a token of appreciation (100 THB, approximately 3 USD at the time of data collection) upon completion.

Following data cleaning, which included removal of incomplete questionnaires and quality screening for response consistency, 378 valid cases were retained for analysis. Of the 22 excluded cases, 14 had more than 20% of items missing and 8 had internal inconsistencies (for example, GPAQ duration responses incompatible with the reported activity domain). Valid sample sizes ranged from 18 to 20 per station, with every station meeting at least 90% of the allocation target. Two stations sat at 18 valid cases rather than 20: these were catchments with high commercial-to-residential ratios where the enumerated household list was exhausted before the quota was met.

The target sample of 400 was determined by two considerations. The first was the operational constraint of budget and surveyor resources. The second was the need to retain a minimum number of outcome events and non-events for multivariable logistic regression [37]. Because several covariates were categorical and therefore contributed more than one model parameter, we report the actual number of non-intercept parameters and the limiting events-per-parameter ratio for each final model in the diagnostic table rather than relying only on a simple variable count.

### 2.3. Variables and Measurement

#### 2.3.1. Outcome Variables

First-mile walking to MRT (RQ1 outcome). Participants were asked whether they walked from their residence to the nearest Pink Line MRT station. Responses were dichotomised (1 = walks; 0 = uses other access mode, including motorcycle taxi, private vehicle, or feeder bus). This variable is the primary outcome for RQ1 and the primary exposure for RQ2. The primary RQ1 analysis retained all residents within the station catchments because the policy question concerns access-mode propensity among the catchment population. Because some residents were not yet current MRT users, two sensitivity analyses were also conducted. First, RQ1 was re-estimated among the 306 respondents who reported current MRT use (81.0% of the analytic sample), so the outcome reflected an observed access-mode choice rather than a hypothetical one. Second, the RQ2 walking–PA models were re-estimated among the same 306 respondents. Among the analytic sample, 71.7% of all respondents and 72.9% of current MRT users reported walking to the station.

Physical activity sufficiency (RQ2 outcome). We measured PA with the Global Physical Activity Questionnaire (GPAQ), a WHO-endorsed instrument validated for population surveillance in diverse cultural contexts [28,34,38,39]. The GPAQ covers three domains: work (occupational), transport (commuting-related walking and cycling to any destination, including but not limited to MRT access), and recreation (leisure-time exercise, including any recreational walking in the neighbourhood or catchment).

We derived two binary outcome variables. The first was overall PA sufficiency (≥150 min/week total MVPA across all three GPAQ domains), the main RQ2 outcome. The threshold is not expected to be met by first-mile walking alone, which typically contributes 5–15 min one way; the analysis tests whether walkers accumulate more total weekly MVPA across all domains. The second was non-transport PA sufficiency (≥150 min/week MVPA from work and recreation only, excluding the transport domain), the tautology sensitivity outcome. Excluding transport PA addresses the potential circularity between walking to MRT (predictor) and transport PA (a component of the main outcome), and also removes the contribution of any other transport walking within the catchment [29].

#### 2.3.2. Exposure Variables

Neighbourhood walkability (NEWS-A). We measured perceived neighbourhood walkability with the Neighbourhood Environment Walkability Scale–Abbreviated (NEWS-A) [18]. Every NEWS-A item asks residents to rate an aspect of their neighbourhood as they experience it, so every subscale captures a subjective resident assessment rather than an objectively audited feature. This distinction matters for interpretation: the predictors below describe perceived neighbourhood conditions, and the associations reported in Section 3 are between perceived environmental features and reported walking, not between objective features and observed behaviour.

The instrument was translated into Thai and contextually adapted following established cross-cultural protocols. Minor changes included rephrasing North American spatial references (e.g., “blocks” to “streets and alleys”) and adjusting destination types to reflect the Thai urban context (e.g., adding temples and open-air markets), in line with previous studies [34,39,40]. Each of the eight subscales was scored on a 1–5 Likert scale, with higher values indicating a more pro-walking perception of the neighbourhood.

The adapted instrument showed good overall internal consistency (Cronbach’s α = 0.86 across all 52 items pooled), comparable to α = 0.83 reported in a prior Bangkok NEWS-A application. Five subscales met or approached the conventional 0.70 threshold: residential density (α = 0.76), land use access (α = 0.68), walking infrastructure (α = 0.85), aesthetics (α = 0.73), and traffic safety (α = 0.78). The 3-item street connectivity (α = 0.35) and 3-item crime safety (α = 0.51) subscales had lower reliability, a pattern consistent with the original NEWS-A validation [18] and reflecting the limited item count per subscale rather than translation effects. Because connectivity falls well below 0.70, any association involving it is treated as exploratory throughout this manuscript and is not used as a basis for substantive conclusions. For RQ1, walkability was examined at two levels of aggregation: the aggregate NEWS-A score (mean of all eight subscales) and the individual subscale scores.

Covariates. Models were adjusted for potential confounders identified from the transit access and walkability literature and previous studies in Bangkok [34,39,40]. The primary covariate set for RQ1 included sex (male/female), age group (18 to 29, 30 to 44, 45 to 59, ≥60 years), marital status (married/not married), income quartile, BMI category (overweight/obese vs. normal weight), vehicle ownership (owns car or motorcycle vs. none), and distance band. The primary covariate set for RQ2 included the RQ1 covariate set plus the aggregate NEWS-A score and occupation category (sedentary/professional, manual/mobile, not working/student); occupation was included because the non-transport PA outcome incorporates work-domain PA, and respondents in manual or mobile occupations could plausibly accumulate sufficient occupational PA independently of transit access. Education level, attitude towards MRT (positive vs. neutral or negative), and non-communicable disease (NCD) status were evaluated in full-covariate sensitivity models. NCD was not included in primary models because only seven respondents reported an NCD, making the estimate unstable. Residential proximity was measured as network distance from the geocoded residential address to the nearest Pink Line station entrance. Distances were categorised into three bands: inner (0 to 200 m), mid (201 to 600 m), and outer (601 to 1000 m; reference category).

### 2.4. Data Analysis

We fitted binary logistic regression models in R version 4.3.0 [41]. Standard errors were clustered at the station level (20 station catchments) using the sandwich package [42]. Spatial analyses, including network isochrone generation and address geocoding, were done in QGIS LTR version 3.34.11-Prizren. Statistical significance was set at *p* < 0.05. Results are reported as adjusted odds ratios (AORs) with 95% confidence intervals (CIs).

Three procedural choices need explanation. First, cluster-robust standard errors with only 20 clusters can under-cover, so we corroborated the four headline coefficients with a wild cluster bootstrap (Rademacher weights at the cluster level, B = 4999 iterations) [37]. Second, the standard likelihood ratio (LR) test for nested models does not adjust for clustering, so the LR test for disaggregation of NEWS-A is reported alongside a cluster-robust Wald test of the joint equality of the eight subscale coefficients. Third, because some final models had a relatively low events-per-parameter ratio, all primary models were also estimated with L1-penalised (Lasso) logistic regression, with the penalty selected by 10-fold cross-validation, as a guard against overfitting. The data analysis plan is summarised in Table 1.

#### 2.4.1. RQ1: Determinants of First-Mile Walking

We fitted two nested binary logistic regression models, with first-mile walking (1 = walks, 0 = other) as the outcome: (1) Model A (aggregate walkability) which includes the aggregate NEWS-A score and the primary covariate set; and (2) Model B (disaggregated walkability), which replaces the aggregate score with the eight individual subscale scores, while retaining the same primary covariates. Full-covariate sensitivity models added education, attitude towards MRT, and NCD status. A further sensitivity analysis re-estimated Models A and B among the 306 current MRT users (responding to a referee request to verify that RQ1 findings are not driven by including non-users in the catchment population).

Model comparison used three criteria: the Akaike Information Criterion (AIC), McFadden’s pseudo-R^2^, and the LR test between nested maximum-likelihood models. Because the LR test does not account for clustering, a cluster-robust Wald test of joint equality of the eight NEWS-A subscale coefficients was reported alongside it. A significant test in either form supports disaggregation. Because the connectivity and crime-safety subscales had lower reliability, an additional sensitivity model excluded both.

To examine whether walkability effects vary by distance, we ran a stratified analysis estimating the association between walking infrastructure quality (NEWS-A Subscale E) and walking within each distance band. The hypothesis was that sidewalk quality matters most outside the immediate station frontage, where the decision to walk is genuinely discretionary.

#### 2.4.2. RQ2: First-Mile Walking and Physical Activity

Binary logistic regression estimated the association between first-mile walking (exposure) and PA sufficiency (outcome), adjusting for the primary covariate set: distance band, vehicle ownership, sex, age group, income quartile, marital status, BMI category, occupation category, and the aggregate NEWS-A score. Two models were estimated. The main model used overall PA sufficiency (≥150 min/week total MVPA from all GPAQ domains) as the outcome. The tautology sensitivity model used non-transport PA sufficiency (≥150 min/week MVPA from work and recreation domains only) as the outcome, with identical covariates. The percentage attenuation in the AOR between the two models was computed as [(AOR_main − AOR_sens)/AOR_main] × 100, and the corresponding attenuation on the log-odds scale was also reported. Both PA models were repeated among current MRT users only as a sensitivity check. Standardised predicted probabilities of meeting each PA threshold were also computed under counterfactual walking conditions, averaging over the observed covariate distribution, so that the magnitude of the walking–PA association could be expressed on the absolute risk-difference scale alongside the odds-ratio estimates.

#### 2.4.3. Robustness and Diagnostic Assessment

All models underwent a standardised robustness battery with seven components. First, station-cluster-robust standard errors were used to account for the stratified station-catchment sampling design. Second, because cluster-robust variance estimators with only 20 clusters can under-cover, a wild cluster bootstrap with Rademacher weights at the cluster level (*B* = 4999) was applied to the four headline coefficients (sidewalk and traffic-safety in RQ1; walking → overall PA and walking → non-transport PA in RQ2), and the cluster-robust Wald test was reported alongside the LR test for the joint equality of the eight NEWS-A subscale coefficients. Third, all primary models were re-estimated using L1-penalised (Lasso) logistic regression with the penalty parameter selected by 10-fold cross-validation, as a guard against overfitting in models with low events-per-parameter ratios. Fourth, multicollinearity was assessed via variance inflation factors (VIF < 5.0 threshold) [43]. Fifth, influential observations were screened using Cook’s distance (*D* > 1.0 threshold) [44]. Sixth, full-covariate sensitivity models tested whether adding education, attitude towards MRT, and NCD status altered the key estimates. Seventh, additional sensitivity models excluded the two low-reliability NEWS-A subscales and restricted both RQ1 and RQ2 to current MRT users.

## 3. Results

### 3.1. Sample Characteristics and Bivariate Comparisons

Of the 378 participants, 271 (71.7%) reported walking to the nearest Pink Line station, while 107 (28.3%) used an alternative access mode. Table 2 presents sample characteristics stratified by walking status. The two groups were similar across all sociodemographic variables: sex (χ^2^ = 2.49, *p* = 0.114), age (*p* = 0.907), marital status (*p* = 0.229), vehicle ownership (*p* = 0.188), and BMI category (*p* = 0.519). Distance band approached but did not reach significance (χ^2^ = 5.47, *p* = 0.065).

By contrast, the two groups differed on both PA outcomes. Walkers were more likely than non-walkers to meet overall PA sufficiency (90.8% vs. 63.6%, χ^2^ = 38.51, *p* < 0.001) and non-transport PA sufficiency (76.8% vs. 57.9%, χ^2^ = 12.39, *p* < 0.001). The absence of significant sociodemographic differences between walkers and non-walkers suggests that the decision to walk was more closely related to environmental and health-related factors than to individual characteristics.

Table 3 presents NEWS-A subscale scores by walking status. Walkers reported significantly higher scores than non-walkers on three subscales: walking infrastructure (E: M = 3.63 vs. 3.29, t = 3.85, *p* < 0.001), traffic safety (G: M = 3.18 vs. 2.75, t = 4.64, *p* < 0.001), and safety from crime (H: M = 3.65 vs. 3.51, t = 2.26, *p* = 0.024). The aggregate NEWS-A score also differed significantly between groups (M = 3.64 vs. 3.50, t = 5.33, *p* < 0.001). Land use access (C) reached marginal significance (*p* = 0.049), while the remaining subscales did not differ between groups.

### 3.2. Determinants of First-Mile Walking to MRT

Table 4 presents the unadjusted and adjusted logistic regression results for the nested models predicting first-mile walking.

In the unadjusted models, the aggregate NEWS-A score predicted walking strongly (OR = 13.72, *p* < 0.001), as did the walking infrastructure (OR = 1.76, *p* < 0.001), traffic-safety (OR = 1.92, *p* < 0.001), and crime-safety (OR = 1.61, *p* = 0.025) subscales. Inner-band proximity (0–200 m) was significant (OR = 2.23, *p* = 0.026); the mid-band was not (OR = 1.38, *p* = 0.199). Vehicle ownership, sex, and marital status were not significant.

After adjustment with station-cluster-robust standard errors, Model B (eight individual subscales) showed that two route-level features carried most of the walkability effect. Moving from the 25th to the 75th percentile on the walking infrastructure subscale raised the adjusted predicted probability of walking from 66.0% to 78.2%, a 12.2-percentage-point absolute difference (AOR = 2.04, 95% CI: 1.41–2.97, *p* < 0.001). The equivalent traffic-safety contrast raised the predicted probability from 64.6% to 79.8%, a 15.2-percentage-point difference (AOR = 2.13, 95% CI: 1.37–3.32, *p* < 0.001).

Three further subscales were positively associated with walking: aesthetics (AOR = 1.54, *p* = 0.023), crime safety (AOR = 1.67, *p* < 0.001), and land use access (AOR = 1.61, *p* = 0.037). Street connectivity was negatively associated (AOR = 0.53, *p* < 0.001), and land use diversity was non-significant.

The aggregate-walkability specification in Model A returned a large but imprecisely estimated coefficient (NEWS-A AOR = 17.74, 95% CI: 5.97–52.66, *p* < 0.001). This confirms the direction of the composite effect, but not its magnitude. The disaggregation in Model B shows that the bulk of this aggregate signal is carried by sidewalk quality and traffic safety. Inner-band residence remained significant in Model B (AOR = 2.01, *p* = 0.021); the mid-band did not (AOR = 1.27, *p* = 0.360). Proximity and route-level walkability therefore operate jointly rather than interchangeably.

Model B outperformed Model A on every fit criterion: AIC fell from 435.0 to 422.3, pseudo-R^2^ rose from 0.096 to 0.156, and the LR test rejected the aggregate specification (χ^2^ = 26.69, df = 7, *p* < 0.001). The cluster-robust Wald test of joint equality of the eight subscale coefficients corroborated this conclusion (χ^2^ = 37.82, df = 7, *p* < 0.001), so the rejection is not an artefact of unclustered LR inference. The wild cluster bootstrap confirmed the asymptotic significance of the two headline subscale coefficients: walking infrastructure (*p* = 0.002) and traffic safety (*p* = 0.002). Collapsing walkability into a single composite therefore obscures information relevant to predicting walking behaviour, and the differential pattern across subscales is not an artefact of having only 20 clusters.

We treat the predicted-probability contrasts as the primary effect-size scale, with the AORs in support. The Lasso (L1-penalised) sensitivity analysis retained walking infrastructure (β = 0.54, AOR = 1.72) and traffic safety (β = 0.62, AOR = 1.86) as the two largest positive coefficients, with street connectivity retained as a negative coefficient (β = −0.39, AOR = 0.68). The direction was stable under shrinkage, but the AOR magnitudes were attenuated, consistent with the events-per-parameter constraint in Model B (5.4 events per parameter). This supports reading the maximum-likelihood AORs as approximate point values rather than precise estimates.

A pre-specified sensitivity analysis re-estimated Models A and B among the 306 current MRT users, responding to a referee request to verify that the RQ1 findings are not driven by including non-users. Among current users, the aggregate NEWS-A association strengthened (Model A AOR = 26.70, 95% CI: 7.25–98.28, *p* < 0.001), and the two route-quality subscales remained the strongest independent predictors (Model B: walking infrastructure AOR = 1.67, *p* = 0.002; traffic safety AOR = 2.26, *p* = 0.001). The negative connectivity coefficient attenuated to non-significance in this restricted sample (AOR = 0.60, *p* = 0.135), reinforcing the cautious interpretation of that subscale. The LR test continued to favour the disaggregated specification (χ^2^ = 15.16, df = 7, *p* = 0.034).

Stratified analysis by distance band showed that the walking infrastructure effect was strongest at intermediate and outer distances: AOR = 2.15 (*p* = 0.015) in the mid band (201–600 m) and AOR = 1.63 (*p* = 0.043) in the outer band (601–1000 m), but non-significant in the inner band (AOR = 1.65, *p* = 0.436).

### 3.3. Tautology Sensitivity Analysis for First-Mile Walking and Physical Activity

Table 5 presents the association between first-mile walking and physical activity (PA) sufficiency, including the tautology sensitivity analysis. After adjustment for the primary covariate set with station-cluster-robust standard errors, the adjusted predicted probability of meeting overall PA guidelines was 91.2% among first-mile walkers and 60.6% among non-walkers, a 30.6-percentage-point absolute difference (wild cluster bootstrap *p* = 0.001; unadjusted OR = 5.64). The corresponding adjusted odds ratio (AOR = 7.36, 95% CI: 3.70–14.67, *p* < 0.001) is reported in Table 5 but is read as an approximate effect-size estimate because the overall PA model has an events-per-parameter ratio of 4.0; the predicted-probability gap is the primary headline. Occupation was included in the primary covariate set because the non-transport PA outcome incorporates work-domain PA; in a parallel specification that omitted occupation, the AOR was 6.90 (95% CI: 3.04–15.68), indicating that occupational composition exerted a small confounding effect that attenuated, rather than inflated, the unadjusted walking–PA association.

The tautology sensitivity analysis excluded transport-domain PA from the outcome to address the potential circularity between first-mile walking (the predictor) and transport PA (a component of the main outcome). Removing transport-domain PA reduced the adjusted predicted-probability gap to 17.5 percentage points (76.4% among walkers, 58.9% among non-walkers; AOR = 2.31, 95% CI: 1.32–4.06, *p* = 0.003), and the result survived the wild cluster bootstrap (*p* = 0.021). The attenuation was 58.0% on the log-odds scale (68.6% on the odds-ratio scale), indicating that a large share of the main association is sensitive to the mechanical overlap between transport walking and the total PA outcome. The remaining 17.5-percentage-point gap indicates that first-mile walkers were also more likely to meet PA guidelines through occupational and recreational activity alone, even after explicitly controlling for occupation category.

The Lasso (L1-penalised) sensitivity analysis confirmed both directional findings: walking was retained as a strongly positive coefficient in both models (overall PA: β = 1.31, AOR = 3.72; non-transport PA: β = 0.57, AOR = 1.76), with most other covariates shrunk to zero. The Lasso AORs are smaller than the maximum-likelihood AORs, consistent with shrinkage of estimates fitted on a sample with only 64 insufficient-PA cases (events-per-parameter ratio = 4.0 in the overall PA model). The direction and qualitative pattern are unchanged. The 30.6-percentage-point and 17.5-percentage-point adjusted contrasts reported above remain the primary effect-size scale for this analysis, with the AOR magnitudes treated as approximate.

### 3.4. Robustness and Diagnostic Assessment

Table 6 summarises the robustness diagnostics across all models. All adjusted models used station-cluster-robust standard errors across 20 clusters. Because cluster-robust variance estimation can under-cover with few clusters, four headline coefficients were also tested with a wild cluster bootstrap (Rademacher weights, B = 4999): walking infrastructure (*p* = 0.002), traffic safety (*p* = 0.002), walking → overall PA (*p* = 0.001), and walking → non-transport PA (*p* = 0.021). Each wild-bootstrap *p*-value supports the corresponding asymptotic conclusion. The cluster-robust Wald test for joint equality of the eight NEWS-A subscales (χ^2^ = 37.82, df = 7, *p* < 0.001) corroborated the LR test (χ^2^ = 26.69, df = 7, *p* < 0.001).

Variance inflation factors were below 1.40 for every predictor in every model, ruling out multicollinearity. No observation exceeded Cook’s D = 1.0 in any model (maximum observed D = 0.027). The L1-penalised (Lasso) sensitivity analysis retained the same headline predictors as the maximum-likelihood models in every case, with shrinkage in AOR magnitudes but no change in coefficient direction.

Because overall PA was highly prevalent (83.1%), the limiting events-per-parameter ratio was driven by the smaller count of insufficient-PA cases (64), not by total sample size. This is the analytic basis for emphasising standardised predicted probabilities alongside AORs in Section 3.2 and Section 3.3.

The tautology sensitivity analysis produced the expected attenuation: the walking–PA AOR fell from 7.36 (overall PA) to 2.31 (non-transport PA), a 68.6% reduction on the odds-ratio scale and 58.0% on the log-odds scale. The non-transport estimate remained significant at *p* = 0.003. The headline estimates also survived three additional checks. Adding education, attitude towards MRT, and NCD status as full-covariate adjustments left the RQ1 effects essentially unchanged (walking infrastructure AOR = 2.07; traffic safety AOR = 2.11) and the RQ2 effects consistent in direction and magnitude. Excluding the low-reliability connectivity and crime-safety subscales gave walking infrastructure AOR = 1.93 and traffic safety AOR = 2.01. Restricting both RQ1 and RQ2 to current MRT users (*n* = 306) gave walking infrastructure AOR = 1.67, traffic safety AOR = 2.26, walking → overall PA AOR = 8.33, and walking → non-transport PA AOR = 2.76, with the aggregate NEWS-A association strengthened (Model A AOR = 26.70).

## 4. Discussion

### 4.1. Summary of Findings

This study examined two questions about first-mile walking along Bangkok’s Pink Line MRT corridor, and produced two main findings.

First, perceived walkability, measured with NEWS-A, was significantly associated with first-mile walking to MRT. Furthermore, once perceived walkability was decomposed into its NEWS-A subscales and station-level clustering was accounted for, two route-level features carried most of the aggregate effect: walking infrastructure (AOR = 2.04, *p* < 0.001) and traffic safety (AOR = 2.13, *p* < 0.001). Second, first-mile walking was strongly associated with meeting overall PA guidelines (AOR = 7.36, *p* < 0.001), and the association remained significant (AOR = 2.31, *p* = 0.003) once transport-domain PA was removed from the outcome. The attenuation was substantial, but the residual association indicates that the walking–PA relationship is not entirely reducible to measurement overlap. All headline coefficients survived a wild cluster bootstrap with Rademacher weights at the cluster level (B = 4999), and the directional pattern was preserved by Lasso (L1-penalised) regression.

Together, these results indicate two things. In a suburban MRT corridor, the station provides a fixed walking destination, and what shapes the decision to walk is whether the route is safe, continuous, and comfortable enough to be used daily. And first-mile walking is associated with PA outcomes that reach beyond the minutes contributed by the walk itself. Each finding is discussed in turn below.

### 4.2. Built Environment Correlates of First-Mile Walking

The aggregate NEWS-A score was statistically significant in our data, but when it was broken into its subdomains, most of that effect was carried by two components: sidewalk quality and traffic safety. Composite walkability indices can therefore obscure which features are actually associated with the behaviour, and in this Bangkok setting the subdomains did not contribute equally to station access.

Similar patterns have been reported in other high-density Asian cities where sidewalk quality is important for first-mile walking. A study in Taipei found that willingness to walk was shaped by route-level conditions such as smoother pavement, removal of obstacles, and shelter from rain rather than by land use planning alone [45]. A recent study in Singapore found that residents’ walking behaviour and the distances walked were closely tied to sidewalk condition and other pedestrian environment features along the route [46]. A Delhi study of metro station users similarly ranked walking infrastructure and safety as the most important attributes influencing walk accessibility [47].

The traffic safety association (AOR = 2.13) is consistent with the conditions pedestrians encounter along the corridor. Bangkok has one of the highest road-traffic fatality rates in the region, and pedestrians account for a substantial share of those fatalities [33]. Perceived traffic safety has been identified as a predictor of walking behaviour in prior empirical work: a longitudinal study of residents in new Perth neighbourhoods reported that perceived safety was significantly associated with transport walking [48], and a Lisbon study of pedestrian perception reported that perceived safety was a significant component of walkability and influenced walking behaviour [49]. In this setting, traffic safety is not only a matter of general street design but a direct constraint on whether a route to the station is usable on foot. Vehicle speeds on collector roads, the absence of signalised crossings at informal desire lines, and motorcycle encroachment onto sidewalks shift the first-mile walk from an inconvenience into an exposure. Respondents who perceived their routes as safer from traffic were therefore more likely to walk, and this held independently of sidewalk infrastructure in the adjusted model, suggesting that the two operate as separable constraints rather than as a single “pedestrian environment” construct.

The two land use subscales behaved differently. Land use diversity (B) showed no independent association with first-mile walking, whereas land use access (C) was significantly associated with walking (AOR = 1.61, *p* = 0.037). The contrast is consistent with the corridor context. Every respondent was already within the catchment of a fixed destination, the MRT station itself, so the marginal value of having a mix of nearby retail or service types for station access is likely to be small; what matters more is whether everyday destinations along the route are within reach on foot. Comparable patterns have been reported where a primary destination dominates local trip generation [50]. The land use access result is therefore best read as capturing functional reach to local services en route, rather than diversity of land uses per se.

Street connectivity, by contrast, was negatively associated with first-mile walking (AOR = 0.53, *p* < 0.001), a finding that runs counter to much of the Western walkability literature. Previous studies have shown that street connectivity predicts transport walking mainly when paired with meaningful destinations and tolerable traffic [51,52]. Along the Pink Line, highly connected grids coincide with busy commercial intersections, multiple turning movements, roadside vendors, and motorcycles using sidewalks as informal lanes. Under these conditions a more connected grid appears to raise exposure to traffic conflicts rather than improve access. This interpretation should be treated cautiously because the connectivity subscale had low internal consistency (α = 0.35). However, the main sidewalk and traffic-safety findings remained significant when the connectivity and crime-safety subscales were excluded, suggesting that the central route-quality conclusion does not depend on the low-reliability subscales. The result argues against generalising Western findings on grid structure to Asian megacity pedestrian environments without qualification, and it adds a cautionary note to walkability indices that treat connectivity as uniformly pro-walking.

Proximity itself also retained an independent association in the adjusted models, with residents of the inner band (0–200 m) about twice as likely to walk as those in the outer band even after the eight walkability subscales were entered (AOR = 2.01, *p* = 0.021). This indicates that proximity and route-level walkability operate jointly rather than interchangeably: living close to a station raises the probability of walking irrespective of route quality, while at greater distances the route itself becomes the binding constraint. The distance-band stratification gives further detail on this pattern. Walking infrastructure quality was significantly associated with walking in the 201–600 m and 601–1000 m bands, but not in the 0–200 m band. This pattern suggests that below about 200 m the trip is short enough that residents walk regardless of route quality, and that beyond that distance route quality becomes more relevant to the decision. The 201–600 m band contains the bulk of pedestrian trips to transit in the international literature [3], and evidence from Seoul indicates that the built environment–walking relationship varies with distance from the station, with the strongest associations outside the immediate station frontage [53]. The implication for investment is that sidewalk and crossing improvements in the middle and outer rings of the catchment, rather than at the immediate station frontage, are likely to have greater behavioural effect, provided the routes are continuous, shaded, and protected from traffic. This is consistent with earlier Bangkok work, which found that residents tolerate shorter distances than Western planning norms assume and that stated walking distances extend when shade is provided [36].

### 4.3. Association Between First-Mile Walking and Physical Activity

The association between first-mile walking and PA sufficiency is consistent with prior work, and is partly expected because walking to transit is itself transport-domain PA. A review of the transit–PA literature estimated that public transport use adds roughly 8–33 min of walking per day [11], and objective-measurement work has reported that transit commuters accumulate more moderate-intensity activity than non-users [54]. A study in the US [55] reported that primary transit users had 7.3 times the adjusted odds of meeting PA recommendations compared with non-users, a figure close to our main-model estimate. Because the PA outcome is total weekly MVPA summed across all three GPAQ domains, the comparison is between walkers and non-walkers in their full weekly activity, not in the walk to the station considered in isolation. The first-mile walk itself accounts for only a fraction of the 150 min; the rest is accumulated through occupational, other utilitarian, and recreational activity, all of which were measured.

The tautology test sharpened this picture. When transport-domain PA was removed from the outcome, the walking coefficient attenuated from AOR = 7.36 to 2.31 but remained statistically significant (*p* = 0.003). This corresponds to a 68.6% reduction on the odds-ratio scale and 58.0% on the log-odds scale. Much of the conventional walking–PA association in these data therefore appears sensitive to the predictor being embedded in the outcome. The remaining association is not explained by that overlap: first-mile walkers were more likely to meet PA guidelines through occupational and recreational activity alone, even after explicitly adjusting for occupation.

Two explanations for the residual association are plausible here. One is selection: the Pink Line is a recently opened suburban corridor where walking to the station is a deliberate choice rather than a default, and those who make that choice may be more active in other domains as well. The other is environmental: a systematic review of transport-related PA identified shorter travel distances, concentrated destinations, street lighting, and transit provision as consistent correlates [31], and these factors may operate across PA domains rather than within transport alone. The cross-sectional design cannot distinguish between them.

The data do not support a causal claim that first-mile walking independently generates non-transport PA. They do support the narrower reading that the walking–PA association in this setting is not purely an artefact of outcome construction. The distinction matters because much of the transit–PA literature reports large associations without testing whether the result depends on including transport PA in the outcome. The tautology sensitivity test is simple to apply wherever GPAQ or similar domain-specific PA data are available, and it allows a clearer reading of what transit-walking research is actually measuring under two outcome definitions: total PA, which includes transport walking, and non-transport PA, which excludes it.

Read alongside the walkability findings, the two results describe a complementary picture of the Pink Line corridor: perceived walking infrastructure quality and perceived traffic safety are associated with who walks, and those who walk remain more likely to meet PA guidelines even after the transport-domain component is removed.

### 4.4. Limitations, Contributions, and Recommendations for Future Studies

Several limitations apply. The cross-sectional design cannot establish causality and leaves open the possibility of residential self-selection, though the recency of the corridor partly mitigates this: the Pink Line opened in 2023, so few current residents could have chosen their address on the basis of a station that did not yet exist when they moved. All measures were self-reported, with the usual risks of recall and desirability bias. Perceived walkability may also diverge from objective sidewalk and crossing conditions. A more specific concern is common-method bias: because the NEWS-A walkability ratings and the walking-behaviour outcome were both reported by the same respondent within the same questionnaire, residents who walk to the station may also rate their routes more favourably on the walking infrastructure and traffic-safety items than residents who do not walk, regardless of the objective state of the route. This bias would tend to inflate the observed associations between perceived walkability and reported walking rather than deflate them. The associations reported in Section 3 should therefore be read as relationships between perceived environmental features and reported walking behaviour, not as environment–behaviour relationships measured against an objective ground truth. Replication using objective audits of sidewalk continuity, crossing provision, and traffic exposure would be required to confirm the magnitudes reported here.

The sample is corridor-specific, drawn from 20 suburban stations on a single MRT line, so the findings may not generalise to underground systems or to more central corridors. The survey was conducted in a single window in November 2025 and did not span seasons, so Bangkok’s heat and monsoon, likely moderators of walking decisions, could not be assessed. The sample was 65.3% female, probably because daytime interviews reached women at home more readily than working-age men. A further analytic consideration is that the high prevalence of PA sufficiency in this sample (83.1%) meant that the limiting events-per-parameter ratio in the overall PA model fell to approximately 4.0 (64 insufficient-PA cases divided by 16 parameters), below the conventional ten-events-per-parameter rule of thumb [37]. To address this, all primary models were corroborated by a wild cluster bootstrap (*B* = 4999) and an L1-penalised (Lasso) regression sensitivity analysis. Both procedures preserved the direction and statistical significance of every headline finding, and standardised predicted probabilities are reported alongside the AORs so that effect sizes are also expressed on the absolute risk-difference scale where the AORs are most sensitive to the small number of non-events. The cluster-robust standard errors used 20 station clusters; because such estimators can under-cover with a small number of clusters, the wild cluster bootstrap was used as the primary inferential check for the four headline coefficients, with consistent results.

Three contributions of this study extend beyond the Bangkok corridor. First, the analysis illustrates the value of decomposing walkability rather than treating it as a single construct. In this setting, perceived walking infrastructure quality and perceived traffic safety carry most of the aggregate effect, and an aggregate index alone would have pointed the policy discussion toward density and mix rather than toward route quality and traffic exposure. The exploratory negative association with connectivity is consistent with this picture but, given its low subscale reliability, is best treated as a hypothesis for replication with objective network data rather than as a substantive finding. Second, the tautology sensitivity test is a simple and replicable analytic step that allows walking–PA research to separate mechanical overlap from residual behavioural association; it is straightforward to apply wherever domain-specific PA data are available, and its use would discipline the large effect estimates commonly reported in transit–PA studies. Third, the distance-band stratification demonstrates that walkability effects are spatially heterogeneous within a station catchment, with perceived walking infrastructure mattering most in the 201–1000 m range rather than at the immediate station frontage; this finding identifies the 201–1000 m ring as a plausible target for future intervention testing of walking infrastructure improvements.

Three recommendations for future studies follow from these findings. First, because the aggregate NEWS-A effect was carried mainly by walking infrastructure and traffic safety, future research should decompose composite walkability indices into their subdomains rather than report a single score, and should pay particular attention to whether connectivity is pro-walking or anti-walking in the local traffic context. Second, because the walking–PA association attenuated by 68.6% on the odds-ratio scale (and 58.0% on the log-odds scale) once transport-domain PA was removed, studies of transit walking and health should routinely report both total and non-transport PA so that mechanical overlap can be separated from residual behavioural association. Third, because the distance-band results showed that route quality matters most outside the immediate station frontage, future studies should stratify analyses by distance band rather than pool the full catchment, and longitudinal or quasi-experimental designs around new station openings would help separate environmental effects from residential self-selection.

## 5. Conclusions

This study examined first-mile walking to MRT stations along Bangkok’s Pink Line corridor, providing evidence on which features of the neighbourhood environment are linked to the decision to walk, and on how much of the walking–physical activity association survives when the circularity between walking as the exposure and transport PA as the outcome is removed. Our findings suggest that the usual focus of walkable urbanism on density, mix, and proximity does not fit well with what residents of a suburban Asian transit corridor actually need in order to walk to a station.

Our findings highlight two main points. First, when aggregate perceived walkability is broken into its NEWS-A subscales, perceived walking infrastructure quality and perceived traffic safety carry the effect, while perceived land use diversity drops out as an independent correlate. Perceived street connectivity showed an exploratory negative association in our data, but the corresponding subscale had low reliability (α = 0.35) and the result is reported as a hypothesis for replication with objective street-network data, not as a substantive contribution. Composite walkability scores can therefore direct policy attention toward density and mix when the binding constraint is the perceived quality and safety of the route itself. Second, first-mile walking is strongly associated with meeting physical activity guidelines, but that association is substantially reduced when transport walking is removed from the total PA outcome. The residual behavioural association persists after transport-domain PA is removed, indicating that first-mile walkers are more likely to be active across other domains as well.

These findings offer evidence-based guidance for urban planners and policymakers in Bangkok and similar tropical megacities. Proximity remains an independent driver of first-mile walking, with residents inside the 200 m frontage about twice as likely to walk as those further out, but proximity alone is not sufficient: investment in continuous sidewalks, shade, and traffic-calming along the 201–1000 m ring of MRT station catchments is likely to do more to shift the behaviour of mid-distance and outer-distance residents than further densification or land use mixing. The distance-band results indicate that route quality matters most at intermediate and outer distances, where the decision to walk is genuinely discretionary, rather than at the immediate station frontage where short trip length already supports walking. Treating walkability as a single composite score can obscure these priorities, and planning assessments for new stations should routinely report subscale-level walkability data.

While these findings reflect Bangkok’s specific climatic and infrastructural conditions, several implications may be transferable to other rapidly urbanising high-density cities in tropical Asia. The emphasis on route-level walking infrastructure, shaded and continuous sidewalks, and protection from traffic is likely relevant wherever transit corridors pass through heterogeneous street environments. Studies in these settings should also routinely report both overall and non-transport PA so that the mechanical and behavioural components of the walking–PA association can be separated. Longitudinal work around new station openings in similar contexts would further strengthen the evidence base.

Taken together, the results support the case for pedestrian-focused investment as part of transit-oriented development, anchored on the two reliable perceived-environment correlates identified here: perceived walking infrastructure quality and perceived traffic safety. First-mile walking in a suburban Asian transit corridor appears to be less a question of how far the station is than of whether the route is perceived to be safe and comfortable enough to be used daily, and the residual walking–physical-activity association persists even after the mechanical overlap between transport walking and transport-domain physical activity is removed.

## Figures and Tables

**Table 1:** Summary of the analytical plan by research question.

Component	RQ1: Determinants of First-Mile Walking	RQ2: Walking and Physical Activity
Outcome	First-mile walking to MRT (1 = walks, 0 = other mode)	(a) Overall PA sufficiency (≥150 min/week MVPA, all GPAQ domains); (b) Non-transport PA sufficiency (work + recreation only)
Primary exposure	NEWS-A walkability (aggregate and disaggregated subscales)	First-mile walking
Models	Model A: aggregate NEWS-A + covariates; Model B: eight NEWS-A subscales + covariates	Main model: overall PA outcome; Sensitivity model: non-transport PA outcome
Covariates	Primary: sex, age group, marital status, income quartile, BMI, vehicle ownership, distance band; full sensitivity: education, attitude towards MRT, NCD	Primary: distance band, vehicle ownership, sex, age group, income quartile, occupation, marital status, BMI, aggregate NEWS-A; full sensitivity: education, attitude towards MRT, NCD
Model comparison	AIC, McFadden’s pseudo-R^2^, LR test (Model B vs. Model A), cluster-robust Wald test (joint equality of subscales)	Percentage attenuation in AOR between main and sensitivity models; standardised predicted probabilities
Supplementary analyses	Stratified analysis of Subscale E (walking infrastructure) by distance band; sensitivity excluding low-reliability subscales; MRT-user-only re-estimation (n = 306)	MRT-user-only sensitivity analysis (n = 306)
Robustness checks	Station-cluster-robust SEs; wild cluster bootstrap (B = 4999, Rademacher); Lasso (L1-penalised) regression; VIF (<5.0); Cook’s distance; full-covariate sensitivity	Station-cluster-robust SEs; wild cluster bootstrap (B = 4999, Rademacher); Lasso (L1-penalised) regression; VIF (<5.0); Cook’s distance; full-covariate and MRT-user-only sensitivity
Software	R 4.3.0 (sandwich, glmnet packages); QGIS 3.34.11-Prizren	R 4.3.0 (sandwich, glmnet packages); QGIS 3.34.11-Prizren

**Table 2:** Sample characteristics by first-mile walking status (n = 378).

Characteristic	Total (n = 378)	Walk (n = 271)	No Walk (n = 107)	χ^2^	*p*
Sex				2.49	0.114
Male	131 (34.7)	101 (37.3)	30 (28.0)		
Female	247 (65.3)	170 (62.7)	77 (72.0)		
Age group				0.55	0.907
18–29 years	68 (18.0)	51 (18.8)	17 (15.9)		
30–44 years	106 (28.0)	74 (27.3)	32 (29.9)		
45–59 years	137 (36.2)	98 (36.2)	39 (36.4)		
≥60 years	67 (17.7)	48 (17.7)	19 (17.8)		
Marital status				1.44	0.229
Married	204 (54.0)	152 (56.1)	52 (48.6)		
Income groups				0.07	0.995
<10,000 THB	103 (27.2)	73 (26.9)	30 (28.0)		
10,000–20,000 THB	93 (24.6)	67 (24.7)	26 (24.3)		
20,001–30,000 THB	98 (25.9)	71 (26.2)	27 (25.2)		
>30,000 THB	84 (22.2)	60 (22.1)	24 (22.4)		
Vehicle ownership				1.74	0.188
Owns vehicle	251 (66.4)	174 (64.2)	77 (72.0)		
Occupation				0.53	0.765
Sedentary/professional	156 (41.3)	111 (41.0)	45 (42.1)		
Manual/mobile	123 (32.5)	91 (33.6)	32 (29.9)		
Not working/student	99 (26.2)	69 (25.5)	30 (28.0)		
BMI category				0.42	0.519
Overweight/obese	172 (45.5)	120 (44.3)	52 (48.6)		
Distance from home				5.47	0.065
0–200 m (inner)	65 (17.2)	53 (19.6)	12 (11.2)		
201–600 m (mid)	149 (39.4)	109 (40.2)	40 (37.4)		
601–1000 m (outer)	164 (43.4)	109 (40.2)	55 (51.4)		
Health outcomes					
PA sufficient (overall)	314 (83.1)	246 (90.8)	68 (63.6)	38.51	<0.001 ***
PA sufficient (non-transport)	270 (71.4)	208 (76.8)	62 (57.9)	12.39	<0.001 ***

Note. Values are n (%). χ^2^ = Pearson’s chi-square. *** *p* < 0.001.

**Table 3:** NEWS-A subscale scores by walking status (*N* = 378).

NEWS-A Subscale	Walk (n = 271) M (SD)	No Walk (n = 107) M (SD)	t	*p*
A: Residential density	3.83 (0.55)	3.87 (0.52)	−0.64	0.524
B: Land use mix—diversity	3.87 (0.61)	3.80 (0.64)	0.93	0.351
C: Land use mix—access	3.96 (0.53)	3.84 (0.53)	1.97	0.049 *
D: Street connectivity	3.61 (0.50)	3.67 (0.47)	−1.02	0.309
E: Walking infrastructure	3.63 (0.74)	3.29 (0.84)	3.85	<0.001 ***
F: Aesthetics	3.42 (0.64)	3.30 (0.64)	1.69	0.092
G: Traffic safety	3.18 (0.84)	2.75 (0.78)	4.64	<0.001 ***
H: Safety from crime	3.65 (0.56)	3.51 (0.54)	2.26	0.024 *
Aggregate NEWS-A	3.64 (0.23)	3.50 (0.24)	5.33	<0.001 ***

Note. Independent-samples *t*-test. * *p* < 0.05, *** *p* < 0.001.

**Table 4:** Unadjusted and adjusted logistic regression models predicting first-mile walking to MRT (*N* = 378).

Variable	OR	95% CI	*p*	AOR	95% CI	*p*
Model A: Aggregate NEWS-A + covariates						
NEWS-A aggregate	13.72	4.87–38.68	<0.001	17.74	5.97–52.66	<0.001 ***
Inner (0–200 m)	2.23	1.10–4.51	0.026	1.84	1.02–3.31	0.044
Mid (201–600 m)	1.38	0.85–2.24	0.199	1.19	0.72–1.97	0.500
AIC = 435.0; Pseudo-R^2^ = 0.096; station-cluster-robust SEs						
Model B: Disaggregated subscales + covariates						
A: Residential density				0.78	0.53–1.14	0.199
B: Land use diversity				1.47	0.89–2.44	0.135
C: Land use access				1.61	1.03–2.53	0.037 *
D: Street connectivity				0.53	0.39–0.72	<0.001 ***
E: Walking infrastructure				2.04	1.41–2.97	<0.001 ***
F: Aesthetics				1.54	1.06–2.24	0.023 *
G: Traffic safety				2.13	1.37–3.32	<0.001 ***
H: Safety from crime				1.67	1.25–2.23	<0.001 ***
Inner (0–200 m)				2.01	1.11–3.65	0.021 *
Mid (201–600 m)				1.27	0.76–2.10	0.360
AIC = 422.3; Pseudo-R^2^ = 0.156; LR test vs. Model A: χ^2^ = 26.69, df = 7, *p* < 0.001						

Note. OR = unadjusted odds ratio; AOR = adjusted odds ratio; CI = confidence interval. Reference: outer band (601–1000 m). Model B replaces aggregate NEWS-A in model A with the eight NEWS-A subscales. Both models include sex, age group, income groups, marital status, vehicle ownership, and BMI. Adjusted CIs and *p*-values use station-cluster-robust standard errors. *** *p* < 0.001, * *p* < 0.05.

**Table 5:** Association between first-mile walking and physical activity: main and tautology sensitivity analyses with occupation in the primary covariate set (*N* = 378).

Variable	AOR	95% CI	*p*
Main model: Overall PA sufficiency (≥150 min/week)			
Walk to MRT	7.36	3.70–14.67	<0.001 ***
Inner (0–200 m)	1.22	0.65–2.30	0.536
Mid (201–600 m)	1.13	0.62–2.06	0.697
NEWS-A aggregate	0.49	0.12–1.96	0.311
AIC = 329.5; Pseudo-R^2^ = 0.144; events 314/378; station-cluster-robust SEs; wild cluster bootstrap *p* = 0.001			
Tautology sensitivity model: Non-transport PA sufficiency			
Walk to MRT	2.31	1.32–4.06	0.003 **
Inner (0–200 m)	1.13	0.57–2.24	0.726
Mid (201–600 m)	0.86	0.50–1.50	0.603
NEWS-A aggregate	1.66	0.55–5.01	0.371
AIC = 464.1; Pseudo-R^2^ = 0.049; events 270/378; AOR attenuation: 7.36 → 2.31 (68.6% OR scale; 58.0% log-odds scale); wild cluster bootstrap *p* = 0.021			

*Note.* Main model outcome: total PA ≥ 150 min/week (all GPAQ domains). Sensitivity outcome: non-transport PA ≥ 150 min/week (work + recreation, excluding transport). Covariates in both models: sex, age group, income groups, marital status, vehicle ownership, BMI, distance band, NEWS-A aggregate, and occupation. Adjusted CIs and *p*-values use station-cluster-robust standard errors. Wild cluster bootstrap *p*-values are based on Rademacher weights at the cluster level with *B* = 4999 iterations. *** *p* < 0.001, ** *p* < 0.01.

**Table 6:** Robustness and diagnostic summary across all models.

Diagnostic	RQ1 Model A	RQ1 Model B	RQ2 Overall PA	RQ2 Non-Transport PA
Station clusters	20	20	20	20
Non-intercept parameters	13	20	16	16
Events/non-events	271/107	271/107	314/64	270/108
Limiting events per parameter	8.2	5.4	4.0	6.8
VIF (all predictors)	max 1.08	max 1.38	max 1.13	max 1.13
Cook’s D > 1.0	None	None	None	None
Max Cook’s D	0.021	0.020	0.027	0.015
AIC	435.0	422.3	329.5	464.1
Pseudo-R^2^	0.096	0.156	0.144	0.049

Note. VIF = variance inflation factor. All values are computed from the corresponding maximum-likelihood specifications reported in Table 4 and Table 5. Cluster-robust standard errors used 20 station clusters; the LR test for Model B vs Model A was χ^2^ = 26.69, df = 7, *p* < 0.001, and the cluster-robust Wald test of joint equality of the eight NEWS-A subscale coefficients was χ^2^ = 37.82, df = 7, *p* < 0.001. Wild cluster bootstrap *p*-values for the four headline coefficients and Lasso direction-retention results are reported in the text.

## Data Availability

The raw data supporting the conclusions of this article will be made available by the authors on request.
